# Short-term monitoring of CFTR modulator therapy in adults and children with cystic fibrosis using low and ultra-low-dose lung CT

**DOI:** 10.1007/s11547-026-02183-3

**Published:** 2026-02-25

**Authors:** Francesca Maccioni, Giuseppe Cimino, Alessandro Longhi, Ludovica Busato, Alessandra Valenti, Lorenza Bottino, Mariateresa Rutigliano, Roberto Alessandrelli, Nicholas Landini, Carlo Catalano

**Affiliations:** 1 Department of Radiological, Oncological and Anatomo-Pathological Sciences, Sapienza University of Rome, Policlinico Umberto I Hospital, Viale del Policlinico 155, 00161 Rome, Italy; 2 Cystic Fibrosis Regional Reference Center, A.O.U. Policlinico Umberto I, Rome, Italy

**Keywords:** Lung, Cystic fibrosis, Computed tomography, Radiation dosage, Children

## Abstract

**Purpose:**

With the advent of CFTR modulators for cystic fibrosis (CF) like Trikafta®, robust, radiation-sparing imaging strategies are urgently needed for monitoring therapeutic effects in both adult and pediatric patients. We investigated whether low-dose and ultra-low-dose lung CT could serve as innovative tool for short-term monitoring of treatment response in adults and children, respectively.

**Methods:**

A total of 30 CF patients (15 adults, 15 paediatric) initiated Trikafta® and underwent baseline CT and 12–18-month follow-up scans. Adults and children were imaged with low- and ultra-low-dose protocols, respectively. Disease severity and extent were quantified using Brody score through both qualitative and quantitative analysis. Pre- and post-treatment CT data were compared (paired t-tests), and results were correlated with spirometry and sweat chloride values.

**Results:**

Remarkably, low and ultra-low-dose protocols maintained high diagnostic quality while reducing radiation exposure (effective dose 2.4 and 0.56 mSv respectively). The Brody score showed significant improvements across all patients, demonstrating substantial decreases in mucous plugging (73%) and bronchial thickening (51%). Each patient exhibited a drop in Brody score after therapy, paralleled by better lung function (*r*_s_ = − 0.71, *p* < 0.0001) and sweat test outcomes (*r*_s_ = 0.7, *p* < 0.0001).

**Conclusions:**

Low- and ultra-low-dose lung CT protocols represent an important advancement in cystic fibrosis imaging, providing sufficient detail to evaluate disease status while substantially reducing cumulative radiation exposure. By enabling reliable short-term assessment of CFTR modulator efficacy through established disease scoring systems, these protocols fill a critical gap in the routine follow-up of patients who require frequent imaging.

## Introduction

Cystic fibrosis (CF) is the most common life-threatening genetic disease among Caucasians, with an incidence of approximately 1 in 3000–4000 live births, as determined by neonatal screening [1]. It is caused by mutations in the CFTR gene, leading to decreased or absent activity of the cystic fibrosis transmembrane conductance regulator (CFTR) protein, a chloride ion transporter critical for airway surface liquid hydration [2–6]. Defective ion transport results in reduced hydration of airway secretions, contributing to hyperviscosity, obstruction, and chronic respiratory complications, the leading cause of CF-related mortality. CF diagnosis is often made at birth through mandatory newborn screening involving immunoreactive trypsin (IRT) assay [7, 8]. Further diagnostic confirmation is achieved via sweat testing, CFTR gene sequencing, and, if necessary, advanced techniques like nasal potential difference (PND) or intestinal current measurement (ICM). Disease progression is characterized by chronic infection, inflammation, and structural lung damage, which can occur even before pulmonary symptoms manifest in childhood [9–11].

Pharmacological therapies for CF have significantly evolved during the last few years, shifting focus from managing symptoms to addressing CFTR protein dysfunction. CFTR modulators, such as Trikafta® (a combination of ivacaftor, tezacaftor, and elexacaftor), represent a breakthrough by targeting structural and functional abnormalities of CFTR, potentially altering disease progression [12–19].

Diagnostic imaging, particularly lung CT, plays a critical role in monitoring CF progression and evaluating treatment efficacy. Over the past two decades, lung CT has largely replaced traditional radiographic methods due to its superior accuracy and detail [20–23]. CT can detect key CF-related lung abnormalities such as consolidations, mucous plugging, bronchial thickening, air trapping, and bronchiectasis [24].

The Brody II is a widely used CT scoring system which focuses on the lobar subdivision of the lungs and evaluates the extent and size of bronchiectasis, mucoid impactions, peri-bronchial thickening, parenchymal consolidations, and air trapping. Brody II has shown great reproducibility, applicability across age and sex, enhanced sensitivity to early alterations, and ability to predict exacerbations [25]. Additionally, it facilitates better correlation between low-dose CT and clinical imaging [26]. Current guidelines recommend routine CT scans every 24 months for asymptomatic patients or more frequently for symptomatic cases, depending on disease severity [27–30].

Recent advances in low-dose and ultra-low-dose CT protocols may enable more frequent imaging with reduced radiation exposure, enhancing monitoring of disease progression [31–34].

Our purpose was to assess the efficacy of low and ultra-low-dose CT in monitoring short-term results (12–18 months) of new CFTR modulators, following changes in imaging parameters during treatment both in pediatric and adult CF patients using the Brody II CT scoring system. Imaging results will be compared with clinical markers, including forced expiratory volume in 1 s (FEV1) and chloride sweat tests (CST).

## Methods

### Patient cohort

This prospective, parallel-group, monocentric study was conducted on patients affected by CF candidates to undergo new therapy with CFTR modulators on the basis of clinical evaluations. Patients displayed a spectrum of disease severities typical for individuals initiating CFTR modulator therapy with Trikafta®.

All selected adult and pediatric patients underwent baseline LDCT and ULDCT respectively, Forced Expiratory Volume in 1 Second (FEV1), and chloride sweat test (CST) prior to and 12–18 months after starting treatment with Trikafta® from February 2020 and January 2023. Data collection and analysis were carried out at the Department of Radiological, Oncological, and Anatomo-Pathological Sciences and the Regional Cystic Fibrosis Centre of the Policlinico Umberto I Hospital, Rome.

Patients were selected among all adult and pediatric population affected by CF that had access to the radiological Department, according to the following criteria.

### Inclusion criteria

1. Access to baseline low-dose (LD) or ultra-low-dose (ULD) CT lung scans.
2. Clinical test data performed for the month corresponding to the basal CT acquisitions.
3. Confirmed CF diagnosis based on gold standard criteria (positive sweat test and CFTR genetic mutation).
4. Eligibility for Trikafta® treatment

### Exclusion criteria

1. Previous exposure to conventional-dose lung CT.
2. Incomplete or out of time range basal clinical test data.

After basal CT and clinical examination, selected patients submitted to Trkafta® treatment were monitored with 12–18-month follow-up LD/ULD CT lung scans, and a parallel clinical evaluation with spirometry and sweat test was performed.

### CT imaging acquisition and reconstruction

Chest CT low- and ultra-low dose protocols were set-up in agreement with the most recent available literature and consensus data [35–40]. Images were acquired using a Philips Ingenuity 64-slice CT scanner with a low-dose technique on inspiration, without contrast medium. Acquisition parameters included a slice thickness of 1 mm, 80–100 kV, a pitch of 1, and a rotation time of 0.4 s, with milliampere adjusted based on patient size.

All CT images were acquired in volumetric scan mode and inspiratory breath-hold with the following parameters: tube voltage of 80–100 kVp; tin filtration enabled; tube current mAs adjusted on patient size; collimation of 1 mm; gantry rotation time of 0.4 s.; pitch of 1. The field of view was tailored to each patient’s requirements. These parameters were chosen to acquire the CT scans at a dose length product (DLP) corresponding to a mean effective dose of 2.4 (LD) and 0.56 (ULD) mSv. CT images were reconstructed both in coronal and transverse orientations at a section thickness (ST) 1 mm and a reconstruction interval (RI) of 0.5 mm, respectively. An iterative reconstruction algorithm was used. All images were transferred and stored to the local PACS system for prospective analysis and reporting.

The CT dose index volume (CTDIvol) and the dose length product (DLP) were recorded for each patient. To allow for intermodal comparison of radiation doses, the respective dose parameters were transformed into effective dose in mSv, which is widely used to compare radiation doses between imaging methods. The effective radiation dose (in millisieverts) was estimated by multiplying the DLP by a chest-specific conversion coefficient (0.014 mSv/mGy × cm).

Low-dose CT protocols were used for adult patients, while ultra-low-dose CT protocols were applied to pediatric patients. A summary of technical parameters utilized for standard, as reported in literature, low- and ultra-low-dose computed tomography protocol is shown in the following (Table 1).

**Table 1:** Parameters utilized for standard, low-dose and ultra-low-dose computed tomography (STD, LD CT and ULD CT respectively)

Parameter	STD CT	LD TC	ULD TC
Instrument	Philips Ingenuity 64-slice CT scanner	Philips Ingenuity 64-slice CT scanner	Philips Ingenuity 64-slice CT scanner
Scan direction	Cranio-caudal	Cranio-caudal	Cranio-caudal
Z-axis extension	Pulmonary apices—costophrenic sinuses	Pulmonary apices—costophrenic sinuses	Pulmonary apices—costophrenic sinuses
Tube voltage (kVp)	120	100	80
Tin filtration	Enabled	Enabled	Enabled
Tube current time product	Automated tube current modulation (reference mAs: 122) CARE DOSE 4D	Average 40 mAs	Average 20 mAs
Tube current modulation CAREDose	Enabled (weak)	Disabled	Disabled
Collimation (mm)	128 × 0.6	128 × 0.6	128 × 0.6
Gantry rotation time	0.5 s	0.4 s	0.4 s
Pitch	1·2	1	1
Slice thickness / reconstruction interval (mm)	0.6/0.6	1/0.5	1/0.5
Field of view (mm)	300	300	300
Pixel matrix	520 × 520	520 × 520	520 × 520
Effective dose vs standard radiography	60-fold	20-fold	5-fold

### Image analysis

*First reading.* FM with respectively 30 years’ experience, assessed the extent and severity of lung disease according to the Brody II scoring system, a validated tool for evaluating CF severity. Score was calculated for all patients as described in the literature [41] independently from clinical data.

*Second reading.* It was performed within 3 months from first reading by three readers, blind of clinical data and previous reading (FM and NL, radiologists with respectively 30- and 10-years’ experience, AL 4th year resident in radiology). Interobserver agreement was calculated. Disagreement was solved on consensus. Intra-observer agreement (FM) between first and second reading was also calculated.

Using Brody II scoring, readers independently re-evaluated both quantitatively and qualitatively bronchiectasis, bronchial wall thickening, consolidations, bullae, air trapping and mucous plugging (considering lingula as a separate lobe) (Table 2).

**Table 2:** Brody II chest CT scoring system

Bronchiectasis score (range 0–12)	=	Extent of bronchiectasis in central lung 0 = none 1 = 1/3 of lobe 2 = 1/3–2/3 of lobe 3 ≥ 2/3 of lobe	+	Extent of bronchiectasis in peripheral lung 0 = none 1 = 1/3 of lobe 2 = 1/3–2/3 of lobe 3 ≥ 2/3 of lobe	x	Average bronchiectasis size multiplier Average multiplier size 0.5 = 0 1 = 1 1.5 = 1.25 2 = 1.5 2.5 = 1.75 3 = 2
Where average bronchiectasis size	=	Size of largest dilated bronchus 1 ≤ 2x 2 = 2x-3x 3 ≥ 3x	+	Average size of dilated bronchi 1 ≤ 2x 2 = 2x-3x 3 ≥ 3x	+	2
Mucous plugging score (range 0–6)	=	Extent of mucous plugging in central lung 0 = none 1 = 1/3 of lobe 2 = 1/3–2/3 of lobe 3 ≥ 2/3 of lobe	+	Extent of mucous plugging in peripheral lung 0 = none 1 = 1/3 of lobe 2 = 1/3–2/3 of lobe 3 ≥ 2/3 of lobe		
Peribronchial thickening score (range 0–9)	=	Extent of peribronchial thickening in central lung 0 = none 1 = 1/3 of lobe 2 = 1/3–2/3 of lobe 3 ≥ 2/3 of lobe	+	Extent of peribronchial thickening in peripheral lung 0 = none 1 = 1/3 of lobe 2 = 1/3–2/3 of lobe 3 ≥ 2/3 of lobe	x	Severity of peribronchial thickening 1 = mild 1.25 = moderate 1.5 = severe
Parenchyma score (range 0–9)	=	Extent of dense parenchymal opacity 0 = none 1 = 1/3 of lobe 2 = 1/3–2/3 of lobe 3 ≥ 2/3 of lobe	+	Extent of ground glass opacity 0 = none 1 = 1/3 of lobe 2 = 1/3–2/3 of lobe 3 ≥ 2/3 of lobe	x	Extent of cysts or bullae 0 = none 1 = 1/3 of lobe 2 = 1/3–2/3 of lobe 3 ≥ 2/3 of lobe
Hyperinflation score (range 1–4.5)	=	Extent of air trapping 0 = none 1 = 1/3 of lobe 2 = 1/3–2/3 of lobe 3 ≥ 2/3 of lobe	x	Appearance of air trapping 1 = subsegmental 1.5 = segmental or larger		

Moreover, image quality was quantified subjectively by the same three radiologists who scored disease severity; they used five indexes after images examination at lung window settings. Image noise, streak artifact and diagnostic acceptability were scored at each of the levels that were imaged by using a five-point scale: 1, unacceptable; 2, minimally acceptable; 3, acceptable; 4, highly acceptable; and 5, excellent. Grade was expressed as mode values.

#### Clinical markers analysis

Clinical biomarkers, including FEV1 and sweat chloride test (CST), were recorded and correlated with CT findings for the same cohort of patients. Data for these markers were obtained from patient admissions to the Regional Cystic Fibrosis Center at Policlinico Umberto I Hospital, Rome, over the 12–18-month period. Information was sourced from paper records and the Camilla® database.

To ensure close temporal correlation between clinical and imaging results, clinical data were collected exclusively for the month corresponding to the relevant CT acquisitions. FEV1 was measured by spirometry using Sensormedics Vmax® 22, while CST was performed using a conductivity method. Following pilocarpine iontophoresis, sweat collection was conducted with the Macroduct® Wescor system, and chloride concentration was measured via potentiostatic coulometry with a chlorometer.

#### Statistical analysis

Statistical analyses were performed using the GraphPad PRISM software (web-based version) and a free available web statistics tool (https://www.socscistatistics.com). Continuous variables are reported as mean ± standard deviation (SD). Density measurements from the low-dose CT series were analyzed via repeated measures, followed by paired t-tests. Statistical significance was set at *p* < 0.05. Correlation between radiologic and clinical parameters were evaluated by Pearson correlation test and expressed as correlation coefficient (*r*_s_). Variability in inter- and intra-observer readings was assessed using Cohen's kappa statistics and expressed as k.

## Results

A total of 30 patients affected by CF and subjected to Trikafta® treatment were analysed. Data coming from 15 paediatric patients (12 females and 3 males, age ranging between 11 and 18 years) and 15 adults (12 females and 3 males, age ranging between 21 and 57 years) (for baseline characteristics see Table 3) were evaluated for CT parameters follow-up as described in the methods section.

**Table 3:** Baseline characteristics of the study cohort

Characteristic	Value
number of patients	30
pediatrics (6–18 y)	15
adults (> 18 y)	15
mean age ± SD	30.15 ± 13.9
gender (Men vs Women)	6 vs 24

The selected patients showed varied disease severities measurable by the Brody II scoring system on lung CT, with baseline values reflecting moderate to severe structural lung abnormalities. This cohort was considered representative for monitoring CF progression under Trikafta® therapy. Low-dose CT protocols were used for adult patients, while ultra-low-dose CT protocols were applied to pediatric patients. CT outputs measured by using the ionization chamber were used to confirm that the displayed CT dose readings on the CT instrument were within acceptable tolerance limits of ± 20%. The low-dose protocol for adults reduced the potential difference to 100 kV with respect to standard CT, with a mean exposure of 41 mAs and CTDIvol of 2.4 mSv. The ultra-low-dose protocol for pediatric patients reduced the potential difference of further 20 kV, with a mean exposure of 20 mAs, achieving an effective dose of 0.56 mSv. This effective dose corresponds to about fivefold dose received performing a standard radiography [37]. Effective doses were reduced by 65%–75% in pediatric patients compared with low-dose standard thin-section CT. Lung doses were inversely related to patient size, with average doses summarized as 0.35/0.56 mGy (ULD-CT) and 1.6/2.4 mGy (LD-CT) for children and adults, respectively.

Regarding image quality, at lung window settings, each of the three qualitative indexes of low dose thin-section CT image quality (diagnostic acceptability, image noise, and streak artifact) was graded. In particular, image noise was graded as 4 in most of patients (n = 10), range 3–5) and 3 for ultra-low dose CT (n = 9, range 2–4). Similarly, diagnostic acceptability was graded between highly acceptable and excellent for low-dose CT (graded as 5, n = 8, range 3–5) but between acceptable and highly acceptable for ULD-CT (graded as 4, n = 8, range 2–5). Streak artifacts were observed more often in protocol ULD- CT than in low-dose images as they were graded as 4 in most of patients (n = 9), range 3–5) and 3 for ultra-low dose CT (n = 9, range 2–4).

**Fig. 1:**
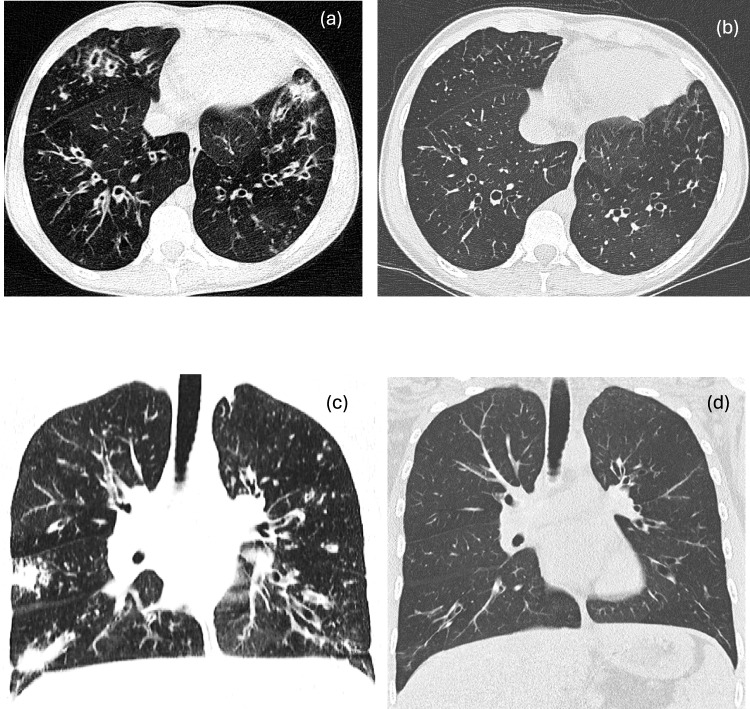
Axial (**a**, **b**) and coronal (**c**, **d**) representative CT-ULD images of 13-year-old female patient prior to initiation of therapy with Trikafta® and after 14 months. A reduction of bronchial wall thickening may be observed (**a**, **b**), consolidations disappeared (**c**, **d**)

**Fig. 2:**
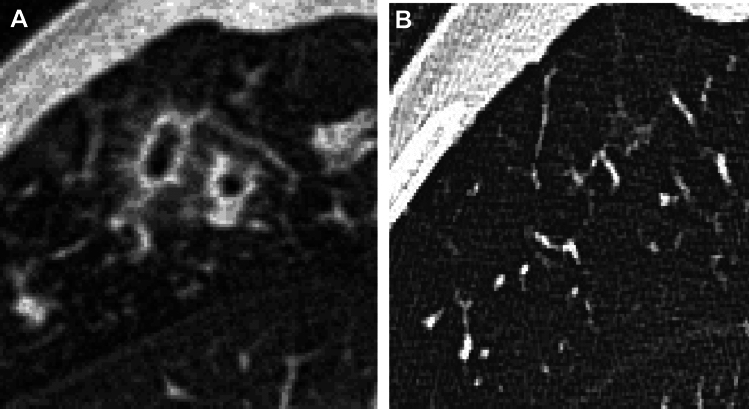
Magnification from Fig. 1**a** and **b** of details showing bronchial thickening and bronchiectasis before and after treatment

**Fig. 3:**
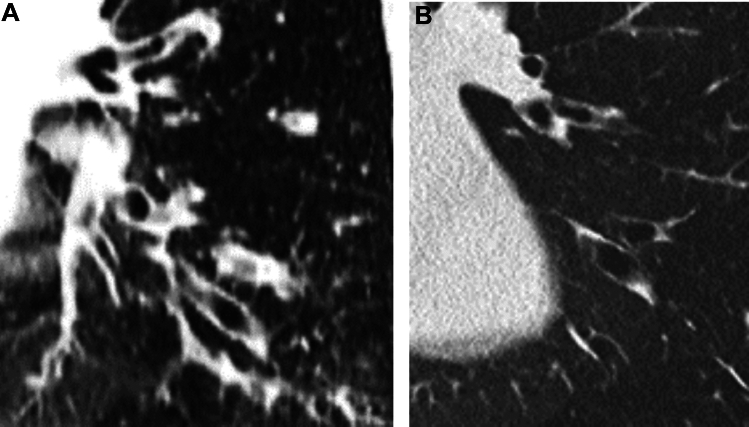
Magnification from Fig. 1**c** and **d** of details showing bronchial thickening and bronchiectasis before and after treatment

Qualitative and quantitative analysis of disease severity parameters reveals that in most of the patients treated with CFTR modulators a significant improvement can be observed both on clinical and radiological tests (Figs. 1, 2 and 3).

**Fig. 4:**
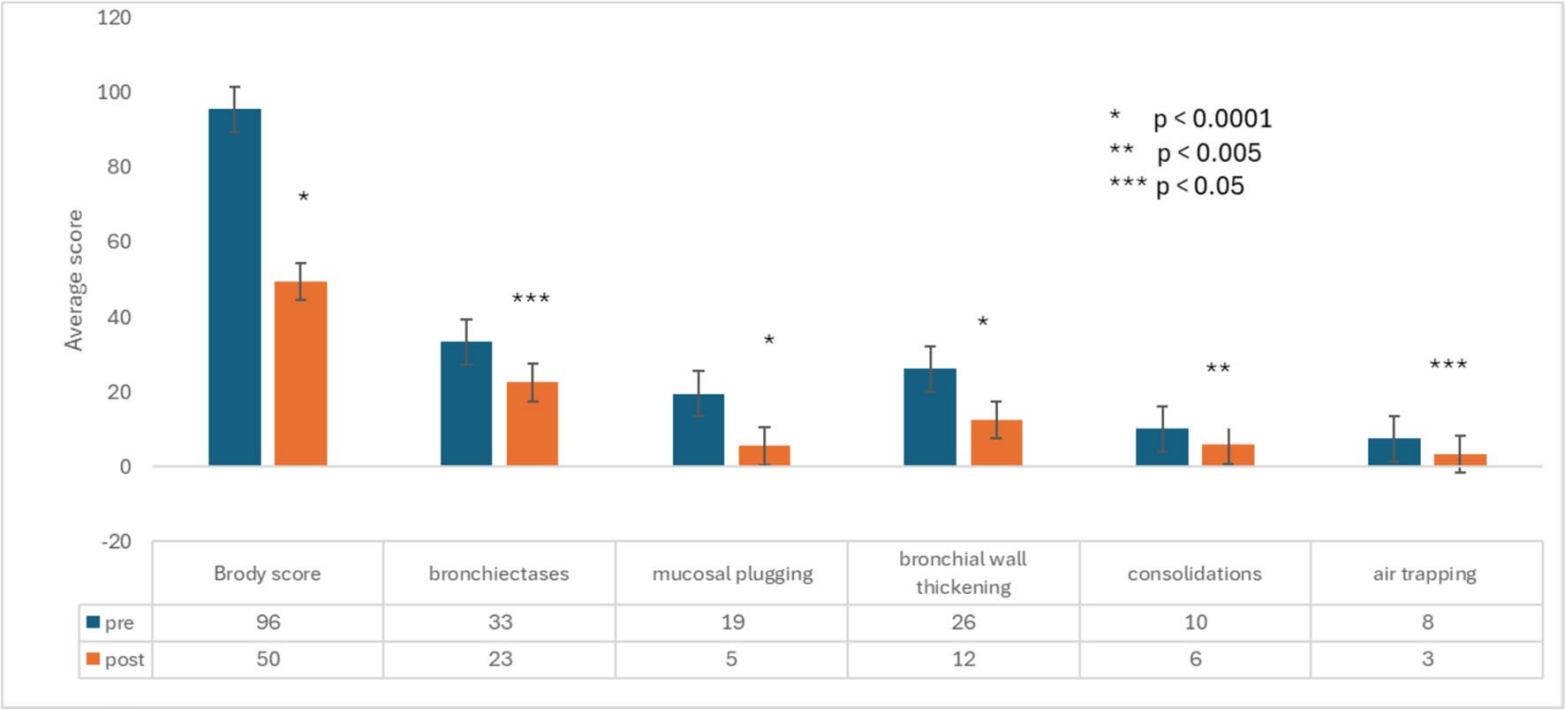
Mean Brody II and single alterations scores with error bars (mean ± SD), with pre- and post-treatment statistically significant differences. Legend: p, *p*-value (paired T-test)

Quantitative analysis of CT images showed a significant improvement in all evaluated parameters after treatment despite the significant low dose of radiations applied (Fig. 4).

Among the parameters, mucous plugging showed the most notable improvement, with the average score reduced from 19 ± 5.5 to 5 ± 4.8 (mean ± SD), representing a 73% reduction (*p* < 0.0001). Bronchial thickening also exhibited a substantial decrease, with the average score dropping from 26 ± 6.3 to 12 ± 3.1, corresponding to a 51% reduction (*p* < 0.0001).

Baseline Brody scores averaged 96 ± 25 indicating significant disease lung involvement. After treatment the average Brody Score showed a global reduction to 50 ± 18 (48% reduction, *p* < 0.0001), reflecting a significant improvement across all patients. However, bronchiectasis and air trapping exhibited comparatively smaller reductions: bronchiectasis scores decreased from 33 ± 11 to 23 ± 13 (34%,* p* < 0.005), and air trapping scores reduced from 7.5 ± 6.7 to 3.3 ± 3.5 (44%, *p* < 0.01). Notably, in pediatric patients, the treatment was particularly effective for bronchiectasis, with scores decreasing from 31 ± 15 to 17.8 ± 5.5 (42%, *p* < 0.005).

Moreover, interobserver agreement was calculated and it showed a substantial correspondence both for LD (k 0.82) and ULD (k 0.70) readings. Also, intra-observer reading showed a significant reproducibility (k 0.73) for all reading data.

**Fig. 5:**
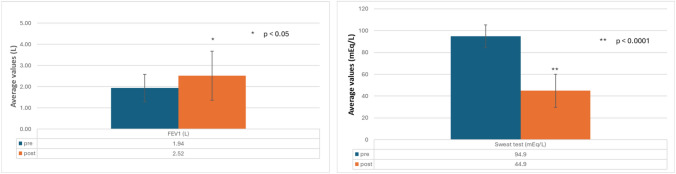
Mean FEV1 (left) and Sweat test (right) values with error bars (mean ± SD), showing pre- and post-treatment statistically significant differences. Legend: p, *p*-value (paired T-test)

Clinical parameter analysis (Fig. 5) further supported these findings. Data evidenced abnormal FEV1 and sweat test values at baseline (1.94 L and 94.9 mEq/mL respectively). After treatment, sweat chloride levels were significantly reduced, dropping from 95 ± 10 mEq/L to 45 ± 15 mEq/L (*p* < 0.0001). Spirometry results (FEV1) showed a significant increase in pulmonary capacity, from 1.94 ± 0.65 L at baseline to 2.52 ± 1.16 L post-treatment (*p* < 0.05).

Moreover, the statistical correlation performed between improvement in Brody score obtained from radiologic parameters and improvement of clinical parameters was significant both FEV1 (*r*_s_ = − 0.71, *p* < 0.0001) and Sweat test (*r*_s_ = 0.7, *p* < 0.0001).

## Discussion

Before the introduction of low-dose protocols, it was neither justified nor feasible to monitor the disease with CT scans at short intervals. In fact, CF is a chronic disease that begins in childhood and lasts a lifetime, making it necessary to keep the cumulative radiation dose within safe limits. Current guidelines recommend routine CT scans every 24–36 months for asymptomatic patients or more frequently for symptomatic cases, depending on disease severity [27–30] The introduction of low-dose protocols and, in particular, ultra-low-dose protocols for children now makes it possible to perform CT scans more frequently while ensuring that exposure to ionizing radiation remains within safe margins [36, 42].

The present study was conducted in order to assess reliability of low- and ultra-low dose CTs. Our lung CT protocols were specifically designed to monitoring CF progression after treatment with new generation CFTR modulators such as Trikafta® with short term controls (12–18 months). Low-dose (2.4 mSv) and ultra-low-dose (0.56 mSv) protocols adopted in the present study are fully compliant with the recommendations developed by the International Commission on Radiological Protection (ICRP). The ICRP recommendations call for a dose constraint of less than 100 mSv per 5 years to minimize the stochastic effects of radiation [43]. The dose of about 100 mSV corresponds to the absorbed radiation dose of about 10 CT total body and the natural background radiation in one year multiplied by 40.

The present study demonstrates that ultra-low-dose and low-dose CT are effective and reproducible methods for monitoring the progression of CF. By maintaining a low radiation dose, more frequent follow-ups were feasible in both young adults and children, enabling an efficient disease surveillance and yielding valuable insights into disease evolution and behavior, as well as into improvements achieved through CFTR modulator therapy.

The analysis of low-dose and ultra-low-dose CT data, comparing pre- and post-treatment average values, revealed significant changes across all CT parameters. Notably, the mean Brody score improved significantly in the short-term follow-ups, in parallel with a decrease in CST values and a corresponding increase in FEV1. Specifically, mucosal plugging and bronchial thickening showed the most pronounced improvements, reflecting a marked decline in secretion retention. This reduction can be attributed to the enhanced fluidization permitted by CFTR modulation achieved through Trikafta®.

On the other hand, although air trapping and bronchiectasis show statistically significant changes, they remain the disease markers least affected by Trikafta® therapy. Both are key anatomopathological, radiographic, and clinical indicators of chronic obstructive damage linked to the pathophysiology of cystic fibrosis and—unlike bronchial wall thickening and mucosal plugging—bronchiectasis typically does not regress after treatment. Likewise, FEV1, as a clinical parameter representing obstructive damage in CF, shows less marked improvement compared to the sweat test. These findings are consistent with reports from several authors who have observed a correlation between bronchiectasis and FEV1 [44, 45]. As noted above, both parameters demonstrated only minimal or no improvement compared to other measures, particularly in adults over 40 years of age. A late initiation of newer treatments, due to the recent availability of CFTR modulators, combined with an already advanced clinical status, may help explain the reduced efficacy of Trikafta® in these patients [46, 47].

Nonetheless, while bronchiectasis improved only slightly in adults as expected, children displayed a significant improvement (*p* < 0.005) after Trikafta® therapy, with several cases showing almost complete regression of bronchiectasis on follow-up CT scans.

Our findings align with previous studies underscoring the higher sensitivity of CT markers compared to FEV1 in detecting both disease progression and treatment effects in CF [48–51]. The Brody II scoring system, selected for its reproducibility and sensitivity [41, 52], further validated the correlation between CT-based measures and clinical outcomes. Other investigations have likewise highlighted the utility of low-dose CT in reducing radiation exposure while maintaining diagnostic accuracy, particularly in pediatric populations [20, 26, 32]. Ultra-low-dose CT for pediatric patients, while achieving radiation doses as low as 0.56 mSv, showed slightly higher image noise and more frequent streak artifacts compared to low-dose CT in adults. Despite this, diagnostic acceptability remained at acceptable to highly acceptable levels. In fact, even if dose reduction may be accompanied by increased image noise and streak artifacts [40] our results suggested that diagnostic reliability is maintained. The trade-off between minimal radiation exposure and minor reduction in image quality is justified given the need for frequent monitoring in children to limit cumulative radiation risk. Ongoing improvements in CT technology and reconstruction algorithms may further mitigate these limitations.

The primary limitation of our study is the small sample size, a consequence of both the relatively recent introduction of CFTR modulator therapies and the inherent rarity of CF. Another limitation concerns the limited representation of the pediatric population aged 6 to 12 years, which—per AIFA, EMA, and FDA guidelines—is eligible for Trikafta® therapy. This reflects insufficient data on treatment outcomes in this age range, largely owing to the recent initiation of therapy, thus preventing a thorough assessment of its long-term efficacy.

Additionally, our single-center design represents another constraint, even though the study was conducted in one of the main regional and national public reference centers for CF research. The relatively small sample size and monocentric design limit generalizability and statistical power. This restricts the ability to capture diverse genetic, phenotypic, and treatment response variability seen in broader CF populations. Future studies including larger cohorts and extended monitoring are planned to understand disease evolution and guide treatment modification over time. In fact, while this study focused on 12–18-month follow-up to capture early treatment effects, we recognize the importance of long-term data to assess the durability of clinical and radiologic improvements—especially for slowly reversible markers like bronchiectasis. We are planning extended longitudinal studies beyond 24 months to evaluate sustained efficacy of CFTR modulators and inform ongoing disease management. Finally, the lack of comparative non–low-dose CT scans constitute a further limitation, as ethical considerations regarding radiation exposure precluded their acquisition.

In conclusion, recently introduced CFTR modulators are reshaping the clinical history of cystic fibrosis (CF). In this context, low-dose and ultra-low-low-dose CT protocols designed to reduce radiation exposure to patients requiring frequent imaging, have proven to be valuable diagnostic tools for monitoring and quantifying treatment efficacy in both adults and children, confirming also the applicability of the Brody score to these CT protocols, for therapeutic response monitoring.

In combination with clinical markers, low- and ultra-low-dose CT protocols currently represent the gold standard for assessing CF therapy with CFTR modulators [28, 31, 53]. By reducing both radiation exposure and the frequency of scans, these protocols significantly lower radiological risk while offering comprehensive, detailed, and—crucially—repeatable follow-up for both adult and pediatric patients. Moreover, our data demonstrates a strong correlation between clinical markers and CT imaging modifications.

## Abbreviations

CF: Cystic fibrosis
CFTR: Cystic fibrosis transmembrane conductance regulator
CT: Computed tomography
FEV1: Forced expiratory volume in 1 s
IRT: Immunoreactive trypsin
PND: Nasal potential difference
ICM: Intestinal current measurement
CST: Chloride sweat tests
LD: Low-dose
ULD: Ultra-low-dose
CTDIvol: Computed tomography dose index-volume
